# Hsa_circ_0004831 serves as a blood-based prognostic biomarker for colorectal cancer and its potentially circRNA-miRNA-mRNA regulatory network construction

**DOI:** 10.1186/s12935-020-01651-8

**Published:** 2020-11-19

**Authors:** Linlin Xing, Mengyan Xia, Xin Jiao, Ling Fan

**Affiliations:** 1 Department of Radiology, Shengjing Hospital of China Medical University, Shenyang, China; 2 Department of Nursing, Shengjing Hospital of China Medical University, Shenyang, China

**Keywords:** Colorectal cancer, CircRNA, Extracellular vesicle, Competitive endogenous RNA, Prognosis

## Abstract

**Background:**

Colorectal cancer (CRC) is a common malignant tumor with unsatisfactory overall prognosis. CircRNAs could be promising prognostic biomarkers in cancers, and play important role in the process of tumorigenesis and progression. Here, we explored the role of hsa_circ_0004831 in blood extracellular vesicles and its prognostic value in CRC.

**Methods:**

The circRNA and mRNA expression level matrix in extracellular vesicles of CRC and normal samples were obtained from the exoRBase database. The corresponding miRNA expression level matrix in extracellular vesicles was downloaded from the BBCancer database. Differentially expressed circRNAs, miRNAs and mRNAs were identified using the limma package of R software at the cut-off criteria of fold change (FC) > 2 and adj. p < 0.05. RT-qPCR assay was conducted to measure hsa_circ_0004831 expression level in CRC blood samples. A circRNA-miRNA-mRNA regulatory network of hsa_circ_0004831 was constructed based on competitive endogenous RNA mechanism and differentially expressed genes. The mRNAs co-expressed with hsa_circ_0004831 were screened at the cut-off criteria of pearson |r| > 0.3 and p < 0.05. Gene set enrichment analysis (GSEA) based on co-expressed mRNAs was used to explore the potential molecular function of hsa_circ_0004831.

**Results:**

Differentially expressed circRNAs, miRNAs and mRNAs were identified and hsa_circ_0004831 had a FC value of 3.92 in CRC blood extracellular vesicles. The RT-qPCR assay showed that the hsa_circ_0004831 was up-regulated in CRC blood samples. The overall survival analysis found that high expression of hsa_circ_0004831 was linked with poorer prognosis. Finally, a circRNA-miRNA-mRNA regulatory network of hsa_circ_0004831 was constructed based on down-regulated miR-4326 and 12 up-regulated mRNAs. GSEA indicated that mRNAs co-expressed with hsa_circ_0004831 were involved in EMT, WNT and p53 signaling pathways.

**Conclusions:**

The study confirmed the up-regulation of hsa_circ_0004831 in CRC, and it may act as a vital prognostic biomarker. The circRNA-miRNA-mRNA regulatory network of hsa_circ_0004831 could be used to uncover the tumorigenesis and progression of CRC.

## Background

The incidence of colorectal cancer (CRC) ranks the third among all human cancers in 2018 and is a common malignant tumor in the clinic [1, 2]. There are kinds of factors which are closely related to CRC incidence, including genetic alteration, diet, lifestyle factors of physical activity and sedentary behavior [3]. Although encouraging advancement in traditional surgery, radio- and chemotherapy, the prognosis of CRC patients is very poor, especially in metastatic patients, with no more than 10% of 5-year survival rates [4]. Therefore, it is necessary to further elucidate the pathophysiological processes that occur in CRC and explore more efficient biomarkers in prognostic prediction.

As a series of novel non-coding RNAs, circular RNAs (circRNAs) are produced from precursor mRNAs backsplicing and have attracted lots of attention in science study [5]. CircRNAs were characterized by a covalently closed-loop structures without 5’ and 3’ terminals and play vital role in the processes of various cancers progression [6, 7]. Extensive studies have revealed the vital importance of circRNAs in human diseases, including cancers [8–10]. Due to the circular structures being resistant to most RNA degradation, circRNAs are abundant in plasma and may present at levels ten-folds higher than those of the corresponding linear mRNA [11]. Taken together, circRNAs have the potential to be effective diagnostic and prognostic biomarkers. However, the roles of a large number of circRNAs in CRC remain unclear.

In our study, we found that hsa_circ_0004831 was up-regulated in the blood of CRC patients. The Kaplan–Meier analysis with log-rank test found that high expression of hsa_circ_0004831 was linked with poorer prognosis. A circRNA-miRNA-mRNA regulatory network of hsa_circ_0004831 was constructed based on competitive endogenous RNA mechanism and differentially expressed genes in CRC. Moreover, GSEA showed that mRNAs co-expressed with hsa_circ_0004831 were involved in EMT, WNT and p53 signaling pathways. These findings indicated that hsa_circ_0004831 participated in important biological process and may be a potent prognostic biomarker for CRC.

## Methods

### Clinical samples

A total of 81 patients diagnosed with CRC at Shengjing Hospital of the China Medical University and 50 healthy volunteers were enrolled in present study. All the blood samples used in this research were collected with complete informed consent from the participants and ethics approval was obtained from the ethics review committee of the Shengjing Hospital of China Medical University before this study. Serum samples were extracted from blood samples after being centrifuged at 3000 rpm, 4 °C for 10 min and were stored at − 80 °C until RNA isolation.

## RNA isolation and RT-qPCR

Blood samples in containers without coagulant were preserved at 4 °C for 4 h to ensure serum separation. Then, serum was stored at − 80 °C until for use after centrifuged for 10 min at 5000 rpm and 3000 rpm, respectively. Total RNA was isolated from serum with TRIzol reagent (Invitrogen, Carlsbad, CA, United States) according to the manufacturer’s protocol. The concentration and quality of total RNA in all samples were detected using NanoDrop ND-1000 spectrophotometer (NanoDrop, Wilmington, DE, United States). The synthesis of cDNA and RT-qPCR reactions were conducted using a reverse transcription kit and the SYBR Green kit (Takara Bio, Dalian, China). Relative gene expression was determined using the ABI 7500 System (Applied Biosystems, USA) with the following qPCR cycling program: 45 cycles including denaturation at 95 °C for 5 s, annealing at 60 °C for 30 s and extension at 72 °C for 30 s. GAPDH was selected as the reference gene. The primers used for qRT-PCR were shown in Table 1. All genes expression levels were quantified using the ΔΔct method.

**Table 1:** The Primers used for qRT-PCR

Name	Sequence
hsa_circ_0004831	
Forward	5′- AAAGAAGAAAGAGCGTGCCG-3′
Reverse	5′- ATGATCATCAGAGGAGGGCG-3′
miR-4326	
RT primer	5′-GTCGTATCCAGTGCAGGGTCCGAGGTATTCGCACTGGATACGACGTCTGG-3′
Forward	5′-GCCCGC TGTTCCTCTGTCTCCC-3′
Reverse	5′-GTGCAGGGTCCGAGGT-3′
ZBED1	
Forward	5′-CCCGGACGAATTCTTCGAAATGGAGAATAAAAGCCTGGAGAG-3′
Reverse	5′-TGCGGATCACTAGTGCTAGCCTACAGGAAGCTGCTGTCCCTAATG-3′
GAPDH	
Forward	5′-ATGGGGAAGGTGAAGGTCG-3′
Reverse	5′-TTACTCCTTGGAGGCCATGTG-3′
U_6_	
Forward	5′-CTCGCTTCGGCAGCACA-3′
Reverse	5′-AACGCTTCACGAATTTGCGT-3′

### Identification of differentially expressed circRNAs, miRNAs and mRNAs

The circRNA and mRNA expression profiles in extracellular vesicles of CRC and normal samples were obtained from the exoRBase database [12]. The corresponding miRNA expression profile in extracellular vesicles was downloaded from the BBCancer database [13]. To ensure the reliability of data processing, the genes which have no expression value in more than half samples were excluded for analysis. The limma package [14] in R was used to identify differentially expressed circRNAs, miRNAs and mRNAs between CRC and normal samples. |Log2FC| > 1 and adj. P value < 0.05 were considered to be statistically significant difference.

### Construction of circRNA-miRNA-mRNA network

The miRNAs which include miRNA binding sites on hsa_circ_0004831 genome sequence were predicted with Circbank [15] database (https://www.circbank.cn). The overlaps of predicted results and down-regulated miRNAs in CRC were regarded as miRNAs which could be regulated by hsa_circ_0004831 through competitive endogenous RNA mechanism. The target genes of miRNAs were predicted by TargetScan v7.1 tool [16]. Similarly, the overlaps of predicted genes and up-regulated mRNAs in CRC were regarded as target genes which involved in competitive endogenous RNA network. Finally, the circRNA-miRNA-mRNA regulatory network was constructed and visualized using Cytoscape v3.7.1 [17].

### GSEA based on co-expressed mRNAs

The mRNAs co-expressed with hsa_circ_0004831 in CRC and normal samples were identified using pearson correlation analysis. Pearson |r| > 0.3 and p value < 0.05 were considered statistically significant. The expression matrix of co-expressed mRNAs was performed for GSEA to explore the biological differences between CRC and normal samples. The hallmark and KEGG [18] subsets in the Molecular Signatures Database (MSigDB) [19] were used as annotated gene sets during GSEA [20].

### Statistical analysis

Statistical analysis used in present study was performed with GraphPad Prism v 7.00 for Windows (GraphPad Software, USA). The differences of genes expression levels between two groups were analyzed by two-tailed Student’s t-test. The overall survival analysis was performed using Kaplan–Meier curves and log-rank test. The optimal cut-off threshold of low or high hsa_circ_0004831 expression was calculated by X-tile [21]. A p value of < 0.05 was considered statistically significant.

## Results

### Differentially expressed circRNAs, miRNAs and mRNAs

**Fig. 1:**
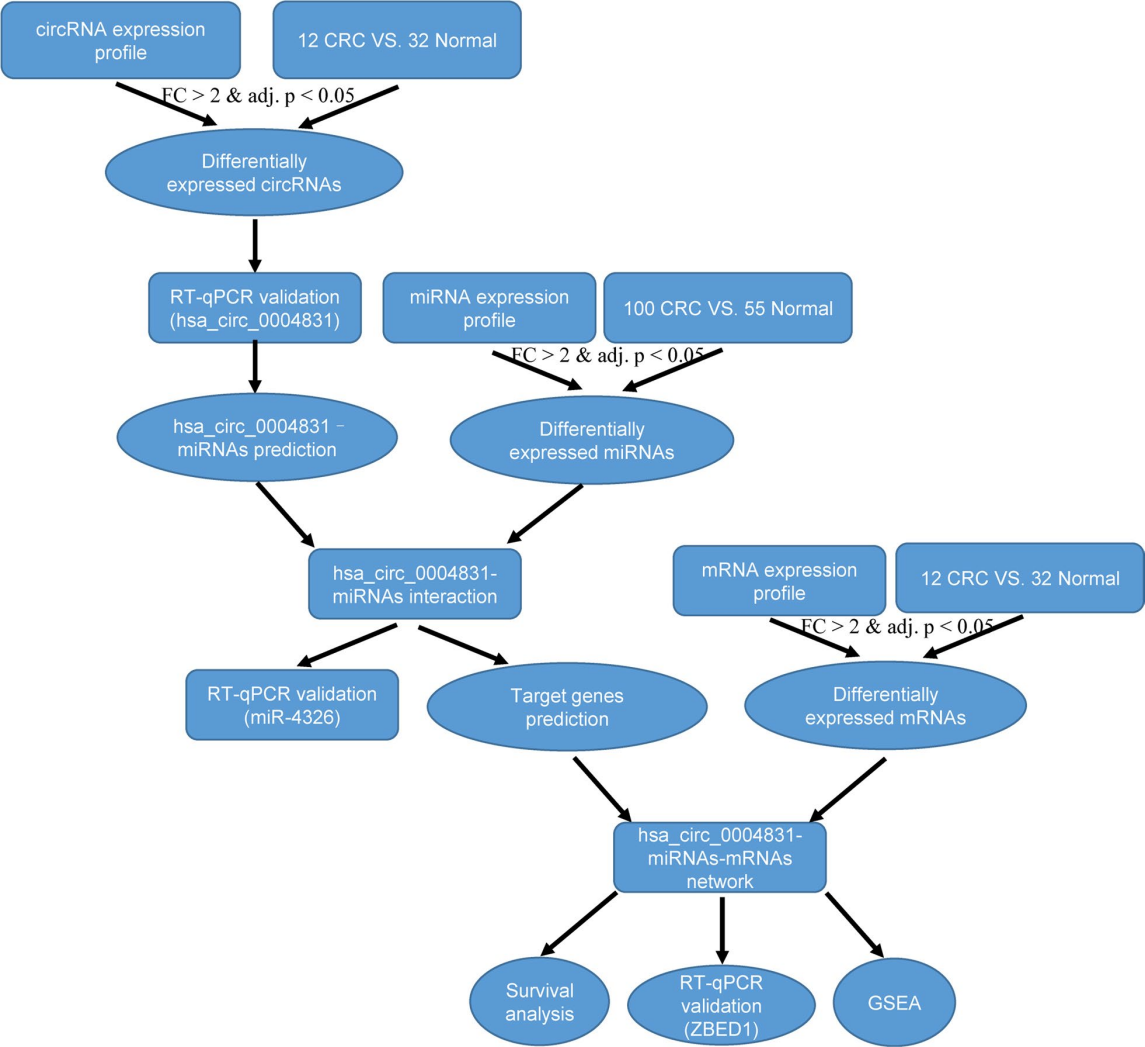
The Flow chart for present study

**Fig. 2:**
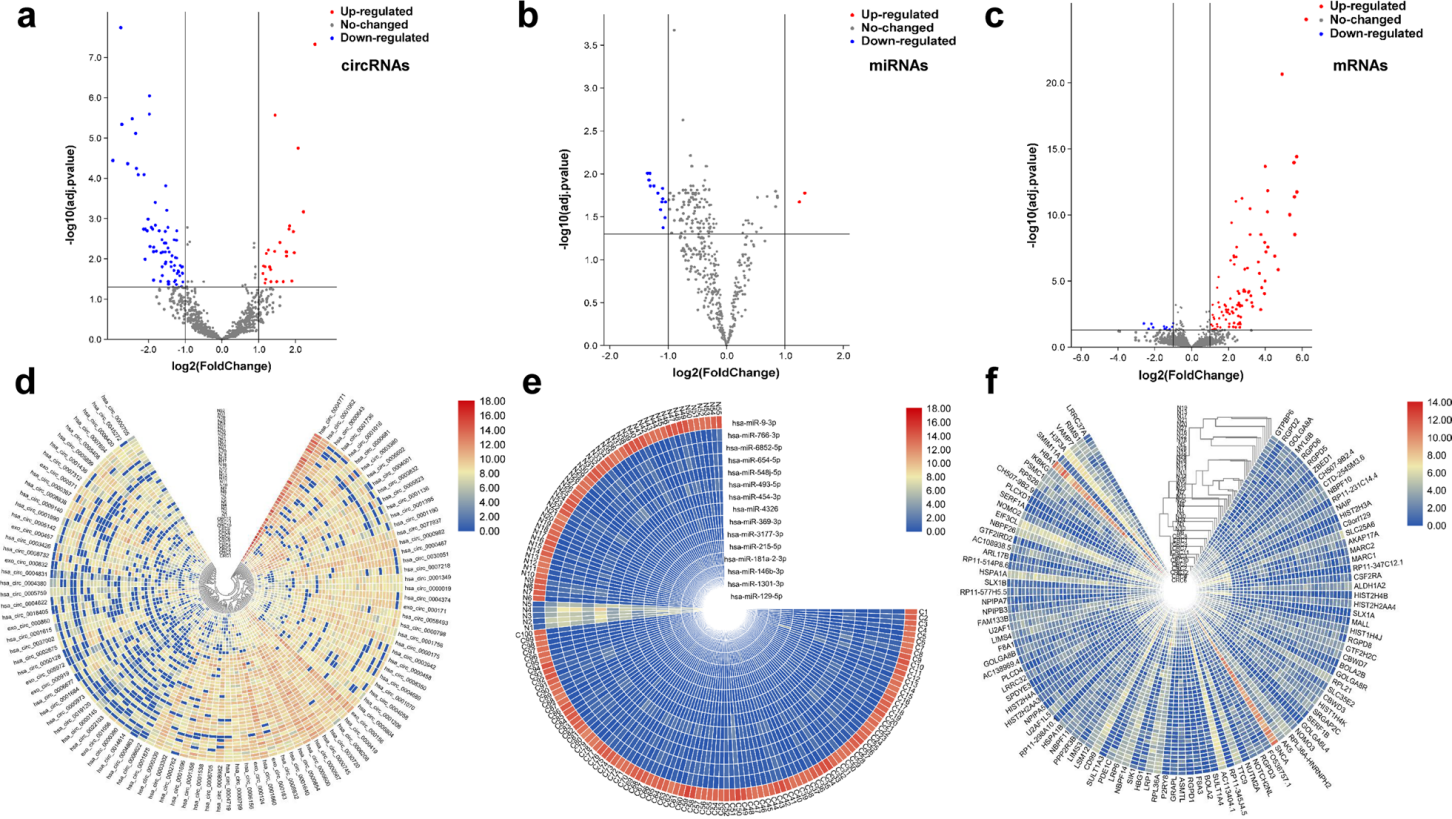
The volcano plots and Heatmaps of differentially expressed circRNAs, miRNAs and mRNAs in CRC. **a**,** d** a total of 28 up- and 73 down-regulated circRNAs were identified from differentially expressed circRNAs. The FC value of hsa_circ_0004831expression in CRC was 3.92. **b**,** e** Two up- and 13 down-regulated miRNAs were identified from differentially expressed miRNAs. The FC value of miR-4326 expression in CRC was 0.47. **c**,** f** Ninety-seven up- and 11 down-regulated mRNAs were identified from differentially expressed mRNAs

The flowchart for this study is shown in Fig. 1. After expression profiles processing, there are 12 CRC and 32 normal samples for circRNA and mRNA expression matrix, and 100 CRC and 55 normal samples for miRNA expression matrix. There are totally 16,115 circRNAs, 1274 miRNAs and 16,605 mRNAs in the expression profiles used for identifying differentially expressed RNAs. According to the cut-off criteria of FC > 2 and adj. p < 0.05, a total of 101 differentially expressed circRNAs (28 up- and 73 down-regulated), 15 differentially expressed miRNAs (2 up- and 13 down-regulated) and 108 differentially expressed mRNAs (97 up- and 11 down-regulated) were identified. The detail results of differentially expression were displayed in the volcano plots and hierarchic cluster heatmaps (Fig. 2). The FC values of hsa_circ_0004831 and miR-4326 expression in CRC were 3.92 and 0.47, respectively.

### hsa_circ_0004831 was up-regulated in CRC and could be a prognostic biomarker

**Fig. 3:**
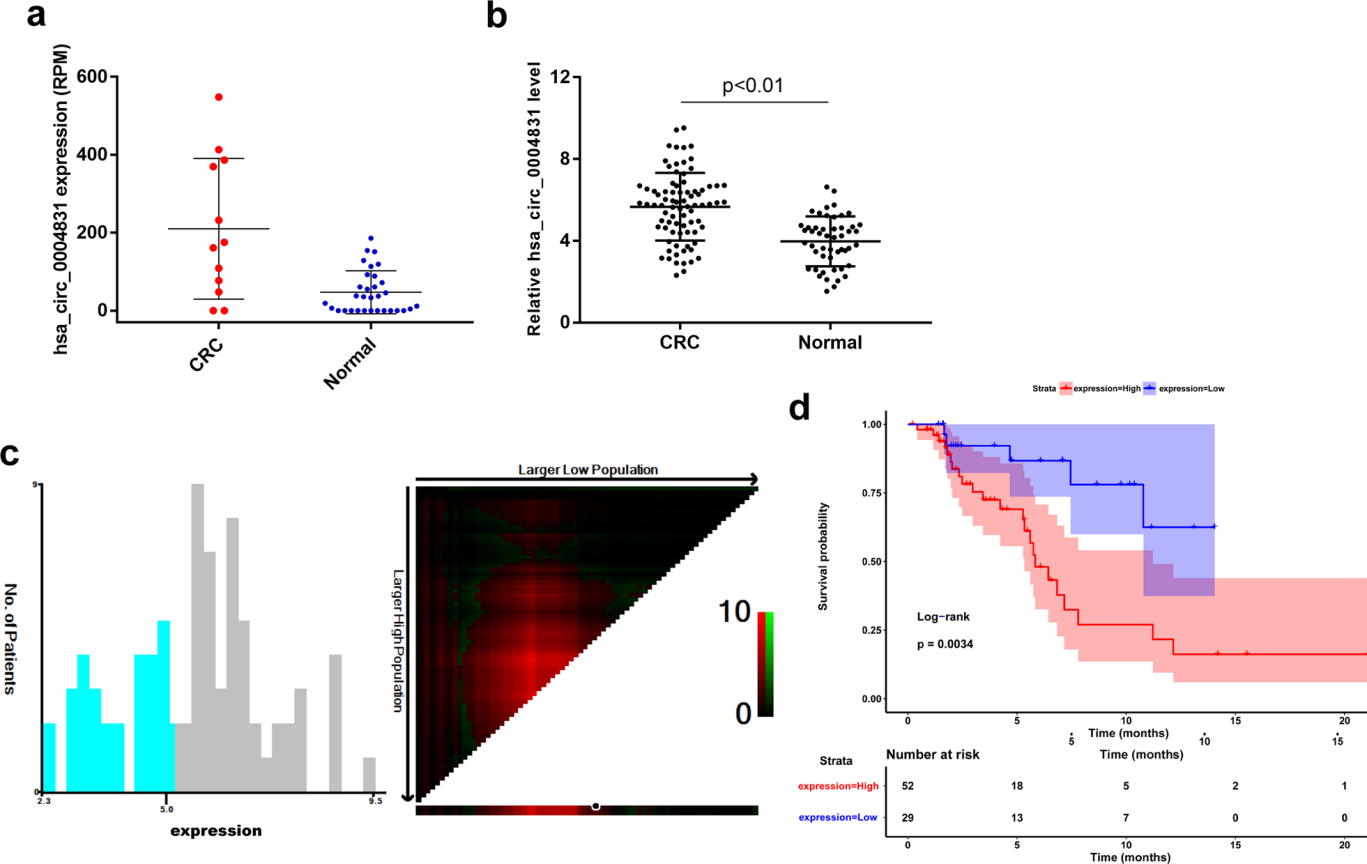
The expression of hsa_circ_0004831 in CRC and survival analysis. **a** Hsa_circ_0004831 was up-regulated in CRC (exoRBase data). **b** Hsa_circ_0004831 was up-regulated in CRC (RT-qPCR data). **c** The optimal cut-off threshold of low or high hsa_circ_0004831 expression calculated by X-tile was 5.1. **d** High hsa_circ_0004831 expression group had a poorer prognosis compared with the low expression group (p = 0.0034)

From circRNA expression profile, we noticed that hsa_circ_0004831 expression level increased 3.92 FC on average in CRC and was significantly up-regulated compared with that in the normal samples (Fig. 3a). Similarly, the RT-qPCR assay showed that hsa_circ_0004831 was significantly up-regulated in 81 CRC blood samples compared with that in 50 healthy volunteers’ samples (Fig. 3b). To investigate the clinical value of hsa_circ_000483, we performed correlation analysis between hsa_circ_0004831 expression and clinicopathological characteristics. The findings suggested that high hsa_circ_0004831 expression was significantly correlated with distant metastasis (p = 0.018) and differentiation grade (p = 0.027) of CRC (Table 2). Then, we explored the role of hsa_circ_0004831 in CRC patients prognostic prediction. The optimal cut-off threshold of low or high hsa_circ_0004831 expression calculated by X-tile was 5.1 (Fig. 3c). The overall survival analysis with Kaplan–Meier and log-rank test showed that patients with high hsa_circ_0004831 (median survival: 5.83) expression had a poorer prognosis than that with low hsa_circ_0004831 (median survival: NA) expression (Fig. 3d). Above findings indicated that hsa_circ_0004831 may be a potent prognostic biomarker for CRC.

**Table 2:** Correlation between hsa_circ_0004831 expression and clinicopathological characteristics

Characteristics	No. of patients	hsa_circ_0004831 (M ± SEM)	t value	p
Age (years)				
≥ 50	45	5.59 ± 0.27	0.37	0.715
< 50	36	5.73 ± 0.25		
Gender				
Male	47	5.50 ± 0.23	1.06	0.291
Female	34	5.89 ± 0.29		
Distant metastasis				
M0	36	5.18 ± 0.28	2.43	0.018*
M1	45	6.06 ± 0.23		
TNM stage				
I + II	33	5.75 ± 0.26	0.36	0.718
III + IV	48	5.61 ± 0.25		
Differentiation grade				
Well/moderate	42	5.28 ± 0.24	2.26	0.027*
Poor	39	6.09 ± 0.26		
Lymph node metastasis				
Negative	35	5.70 ± 0.27	0.15	0.880
Positive	46	5.64 ± 0.25		

* p < 0.05 was considered statistically significant

### The circRNA-miRNA-mRNA regulatory network of hsa_circ_0004831

**Fig. 4:**
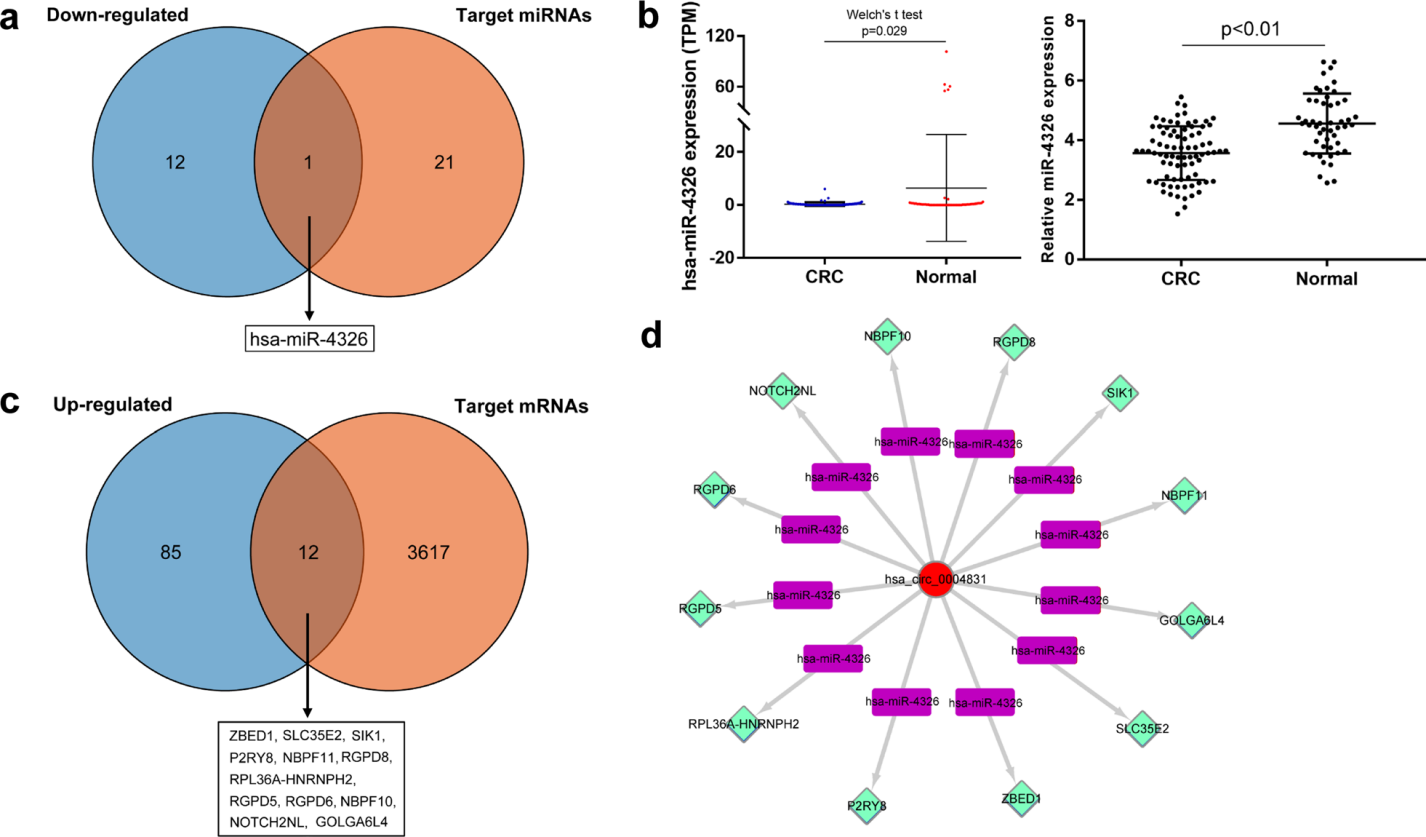
The circRNA-miRNA-mRNA regulatory network construction of hsa_circ_0004831. **a** One miRNA (hsa-miR-4326) was identified to be regulated by hsa_circ_0004831 through competitive endogenous RNA mechanism. **b** Hsa-miR-4326 was down-regulated in CRC (exoRBase data and RT-qPCR assay). **c** Twelve mRNAs were identified as target genes which involved in competitive endogenous RNA network. **d** CircRNA-miRNA-mRNA regulatory network of hsa_circ_0004831 in CRC

Firstly, we obtained 22 miRNAs which include miRNA binding sites on hsa_circ_0004831 genome sequence from Circbank database. After integrating with 13 down-regulated miRNAs, hsa-miR-4326 was identified to be regulated by hsa_circ_0004831 through competitive endogenous RNA mechanism (Fig. 4a). The miRNA expression profile showed that hsa-miR-4326 was significantly down-regulated in CRC compared with that in the normal samples. Besides, RT-qPCR assay confirmed that miR-4326 was down-regulated in 81 CRC blood samples compared with that in 50 healthy volunteers’ samples (Fig. 4b). Then, we predicted 3629 target genes of hsa-miR-4326 from TargetScan. Using similar methods, a total of 12 mRNAs were identified as target genes which involved in competitive endogenous RNA network (Fig. 4c). Thus, a circRNA-miRNA-mRNA regulatory network involved in hsa_circ_0004831, hsa-miR-4326 and 12 mRNAs was visualized using Cytoscape 3.7.1 (Fig. 4d).

### GSEA based on co-expressed mRNAs

**Fig. 5:**
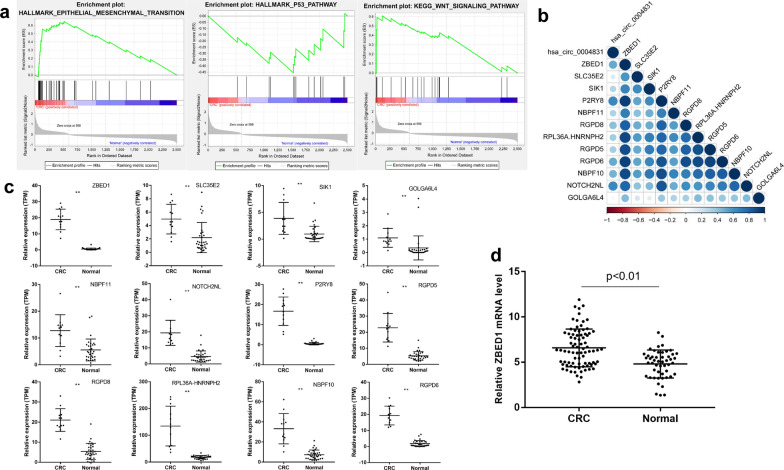
GSEA and identification of mRNAs co-expressed with hsa_circ_0004831. **a** GSEA based on the expression matrix of co-expressed mRNAs showed that hsa_circ_0004831 were involved in EMT, WNT and p53 signaling pathways. **b** pearson expression correlation showed that the expression of hsa_circ_0004831 was positively correlated with that of 12 target mRNAs. **c** The distribution plots of 12 target mRNAs expression in CRC and the normal samples from mRNA expression profiles. **d** RT-qPCR assay indicated that ZBED1 expression was up-regulated in 81 CRC blood samples

In order to further study the functional pathways which hsa_circ_0004831 may participate in, we first obtained mRNAs co-expressed with hsa_circ_0004831 in CRC and normal samples using pearson correlation analysis. At the conditions of pearson |r| > 0.3 and p value < 0.05, a total of 2503 co-expressed mRNAs were identified. GSEA based on the expression matrix of co-expressed mRNAs showed that hsa_circ_0004831 were involved in EMT, WNT and p53 signaling pathways (Fig. 5a). Furthermore, we visualized the pearson expression correlation of hsa_circ_0004831 and 12 target mRNAs. The results showed that the expression of hsa_circ_0004831 was positively correlated with that of target mRNAs (Fig. 5b). The mRNA expression profile indicated that 12 target mRNAs were all significantly up-regulated in CRC compared with that in the normal samples (Fig. 5c). Meanwhile, the mRNA expression level of ZBED1 was detected by RT-qPCR assay. Compared with 50 healthy volunteers’ samples, ZBED1 was found to be up-regulated in 81 CRC blood samples (Fig. 5d).

## Discussion

Mounting studies showed that circRNAs are closely related to CRC carcinogenesis [22–24]. The circRNA-miRNA-mRNA competitive endogenous RNA network plays a critical role in CRC incidence, providing novel insights into the pathogenesis of CRC. The present study identified differentially expressed circRNAs, miRNAs and mRNAs between CRC and normal blood samples. We found that hsa_circ_0004831 expression level increased 3.92 FC on average in CRC. However, the role of hsa_circ_0004831 in CRC are remain unclear. The RT-qPCR assay also confirmed that hsa_circ_0004831 was significantly up-regulated in 81 CRC blood samples compared with that in 50 healthy volunteers’ samples. All these results revealed that hsa_circ_0004831 may act as an oncogene in CRC.

Hsa_circ_0004831 is a transcription product of the remodeling and spacing factor 1 (RSF1) gene located on chromosome 11. The potential function of hsa_circ_0004831 in the tumorigenesis and progression of CRC attracted our interest. The Kaplan–Meier analysis with log-rank test found that high expression of hsa_circ_0004831 was linked with poorer prognosis. Due to the stable existence of circRNAs in plasma and favorable patient compliance, hsa_circ_0004831 may be a potent prognostic biomarker for CRC.

Present evidences showed that circRNAs can act as competitive endogenous RNAs to reduce the miRNAs expression levels and release their targeted inhibition to mRNAs, thereby mediating the expression of protein-coding genes [25–27]. Lin et al. found that circCYFIP2 may act as an oncogenic circRNA to promote gastric cancer progression through miR-1205/E2F1 axis and could be a potent therapeutic target for gastric cancer [28]. Hsa_circ_0006168 was found to be significantly increased in the tissue and cell line level of esophageal squamous cell cancer and may regulate the expression of mTOR via sponging miRNA-100 [29]. Besides, Su et al. found that hsa_circ_0070269 can play a vital role in hepatocellular carcinoma progression through sponging miR-182 and thus increasing NPTX1 expression [30]. However, the competitive endogenous RNA mechanism of hsa_circ_0004831 in CRC is still unclear. Thus, we constructed a circRNA-miRNA-mRNA regulatory network of hsa_circ_0004831 based on competitive endogenous RNA mechanism and differentially expressed genes in CRC. The findings can provide novel insights into the pathogenesis of CRC.

To further investigate the biological pathways which hsa_circ_0004831 may participate in, we performed GSEA using the expression matrix of mRNAs co-expressed with hsa_circ_0004831 in CRC and normal samples. The findings of GSEA showed that hsa_circ_0004831 can participate in EMT, WNT and p53 signaling pathways. EMT is a common biological pathway that is necessary for cancer progression since it converts immobile epithelial cells into active mesenchymal cells and thereby promotes metastasis [31]. For example, a previous study demonstrated that silencing circ_0001946 can suppress CRC cell growth, migration, and invasion through EMT pathway inhibition [32]. Ma et al. found that circ5615 may function as competing endogenous RNA of miR-149-5p and activate Wnt/β-catenin pathway to promote CRC progression [33]. Moreover, Li et al. first identified that circRNA CBL.11/miR-6778-5p/YWHAE axis together with the p53 pathway can regulate apoptosis and proliferation of CRC cells [34]. To the best of our knowledge, the role of hsa_circ_0004831 associated with EMT, WNT and p53 signaling pathways in CRC was undefined. Above all, the present study suggested that hsa_circ_0004831 participated in vital cancer-related biological process in CRC and provided novel target into the treatment of CRC.

However, there were also several drawbacks in present study. The study design only confirmed genes expression level and was short of findings from in vivo/vitro experiments. Even so, the subject matter of present study can be of interest to researchers in the field, and we will conduct in-depth study in further explorations. Besides, we selected TRIZOL reagent to extract RNA from serum samples, which may be not an optimal protocol. At least, the concentration and quality of total RNA in all samples were guaranteed, and we will also improve the relevant protocols in future study.

## Conclusions

This study identified differentially expressed circRNAs, miRNAs and mRNAs in CRC, and RT-qPCR confirmed the up-regulation of hsa_circ_0004831 in CRC. The Kaplan–Meier analysis with log-rank test found that high expression of hsa_circ_0004831 was linked with poorer prognosis. The correlation analysis between hsa_circ_0004831 expression and clinicopathological characteristics suggested that high hsa_circ_0004831 expression was significantly correlated with distant metastasis (p = 0.018) and differentiation grade (p = 0.027). A circRNA-miRNA-mRNA regulatory network of hsa_circ_0004831 was constructed based on competitive endogenous RNA mechanism and differentially expressed genes in CRC. Moreover, GSEA showed that mRNAs co-expressed with hsa_circ_0004831 were involved in EMT, WNT and p53 signaling pathways. These findings indicated that hsa_circ_0004831 participated in important biological process and may be a potent prognostic biomarker for CRC.

## Data Availability

The datasets used and/or analysed during the current study are available from the corresponding author on reasonable request.

## Abbreviations

CRC: Colorectal cancer
FC: Fold change
RT-qPCR: Real time**-**quantitative polymerase chain reaction
GSEA: Gene set enrichment analysis
EMT: Epithelial-mesenchymal transition
CircRNAs: Circular RNAs
KEGG: Kyoto Encyclopaedia of Genes and Genomes
RPM: Reads per million mapped reads
NA: Not available
Adj: Adjusted
